# Paxillin: a crossroad in pathological cell migration

**DOI:** 10.1186/s13045-017-0418-y

**Published:** 2017-02-18

**Authors:** Ana María López-Colomé, Irene Lee-Rivera, Regina Benavides-Hidalgo, Edith López

**Affiliations:** Instituto de Fisiología Celular, Universidad Nacional Autónoma de México, Apartado Postal 70-253, Ciudad Universitaria, México, 04510 D.F. Mexico

**Keywords:** Cancer, Focal adhesions, Cell migration, Signal transduction

## Abstract

Paxilllin is a multifunctional and multidomain focal adhesion adapter protein which serves an important scaffolding role at focal adhesions by recruiting structural and signaling molecules involved in cell movement and migration, when phosphorylated on specific Tyr and Ser residues. Upon integrin engagement with extracellular matrix, paxillin is phosphorylated at Tyr31, Tyr118, Ser188, and Ser190, activating numerous signaling cascades which promote cell migration, indicating that the regulation of adhesion dynamics is under the control of a complex display of signaling mechanisms. Among them, paxillin disassembly from focal adhesions induced by extracellular regulated kinase (ERK)-mediated phosphorylation of serines 106, 231, and 290 as well as the binding of the phosphatase PEST to paxillin have been shown to play a key role in cell migration. Paxillin also coordinates the spatiotemporal activation of signaling molecules, including Cdc42, Rac1, and RhoA GTPases, by recruiting GEFs, GAPs, and GITs to focal adhesions. As a major participant in the regulation of cell movement, paxillin plays distinct roles in specific tissues and developmental stages and is involved in immune response, epithelial morphogenesis, and embryonic development. Importantly, paxillin is also an essential player in pathological conditions including oxidative stress, inflammation, endothelial cell barrier dysfunction, and cancer development and metastasis.

**Electronic supplementary material:**

The online version of this article (doi:10.1186/s13045-017-0418-y) contains supplementary material, which is available to authorized users.

## Background

**Fig. 1:**
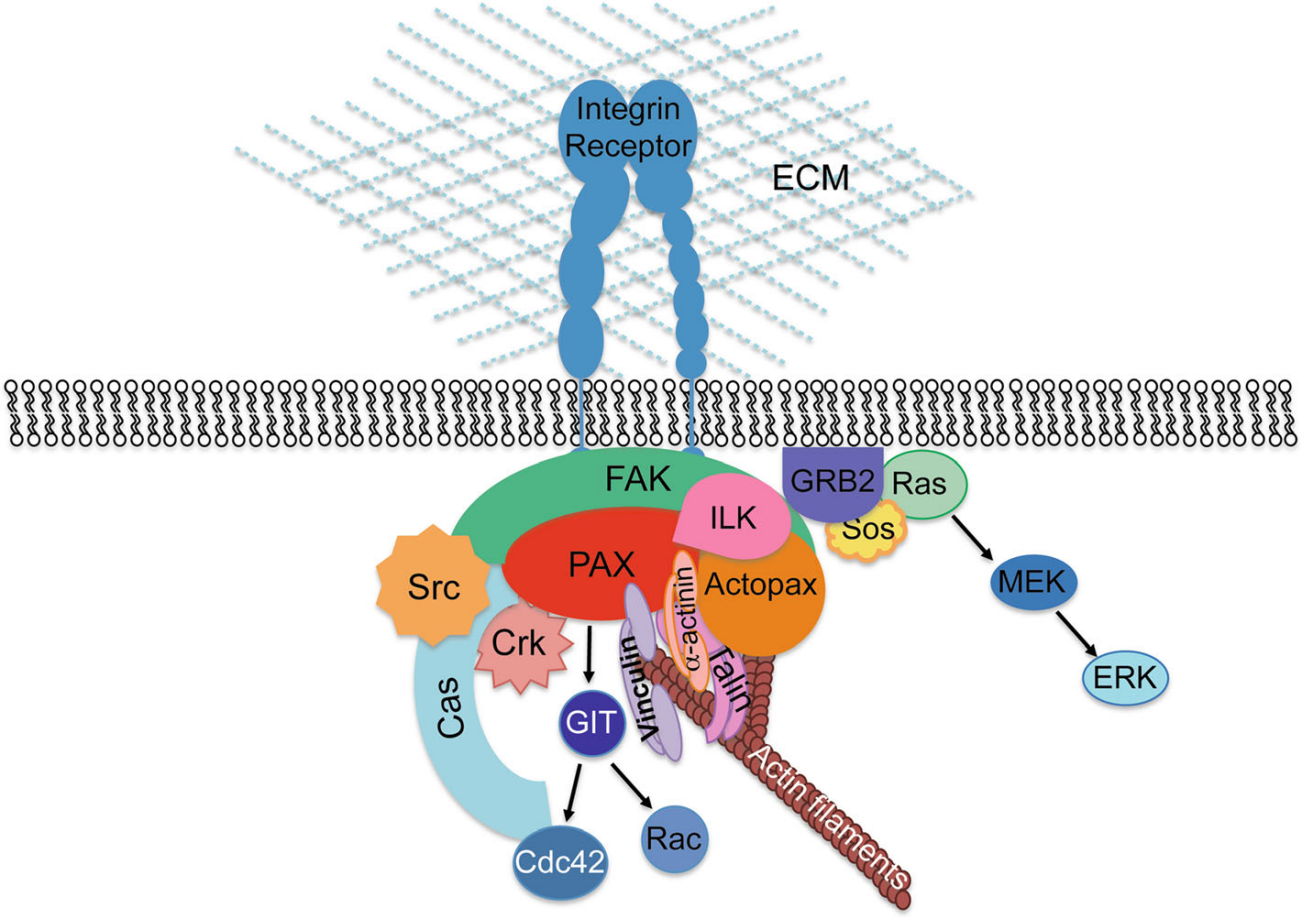
Paxillin at focal adhesions. As a major component of FAs, the phosphorylation of paxillin by FAK upon integrin activation allows the recruitment of several enzymes and structural molecules. Dynamics of paxillin association with these molecules results in changes in cell movement and migration. The figure depicts a simplified scheme showing the main components of a focal adhesion complex. The number and composition of the FA complex and the related signaling pathways may vary in different scenarios, according to the subunit composition of integrin receptors and the specific signaling pathways activated by distinct combinations, in addition to the type and developmental stage of the cell [44]

Paxillin is a main component of focal adhesions (FAs) and plays an important role in the transduction of extracellular signals into intracellular responses, triggered by the engagement of integrins with the extracellular matrix (ECM). As a scaffolding protein, paxillin contributes to the recruitment of specific kinases and phosphatases, cofactors, oncoproteins, and structural proteins, involved in intracellular signaling cascades (Fig. 1). The activation of these pathways ultimately leads to the reorganization of the actin cytoskeleton and the assembly/disassembly of focal adhesions (FAs) required for cell attachment, spreading, and migration [1]. Paxillin is not only recruited at nascent FAs at the cell front for the assembly of adhesion complexes but also required for the disassembly of FAs at the rear end of the cell during cell movement and migration [2, 3]. Hence, paxillin may exert positive or negative effects on cell migration [4]. Although paxillin is mainly localized at FAs, it is also present at cytoplasmic and nuclear locations, where it may affect gene transcription, thus acting as a direct link from the plasma membrane and the cytoskeleton to the nucleus [5]. In spite of its inclusion in protein complexes with cytoskeletal proteins and enzymes, paxillin does not exhibit enzyme activity but provides docking sites for other proteins to facilitate the assembly of multiprotein complexes.

## The paxillin family

The paxillin family genes include paxillin (PXN), Hic-5 (TGFB1I1), and leupaxin (LPXN), which share binding sequences for several interacting proteins but differ in distribution and specific functions [6]. Paxillin is expressed in most tissues analyzed, with the lowest level of expression found in the nervous system. In contrast, Hic-5 expression is restricted: whereas epithelial cells do not express Hic-5, this protein is highly expressed in the smooth muscle, particularly in the vascular muscle. As a functional difference between these homologous proteins, whereas the lack of expression of paxillin in embryonic development is lethal, the deletion of Hic-5 results in minor alterations in the vascular system development. Although leupaxin expression was suggested to be restricted to leukocyte cell lineage, leupaxin was recently identified in cells from diverse lineages. Regarding functional relations among paxillin family members, the increase in Hic-5 or ectopic expression of leupaxin has been shown to prevent paxillin phosphorylation and its direct interaction with proteins involved in downstream signaling [6, 7].

### The paxillin gene

**Fig. 2:**
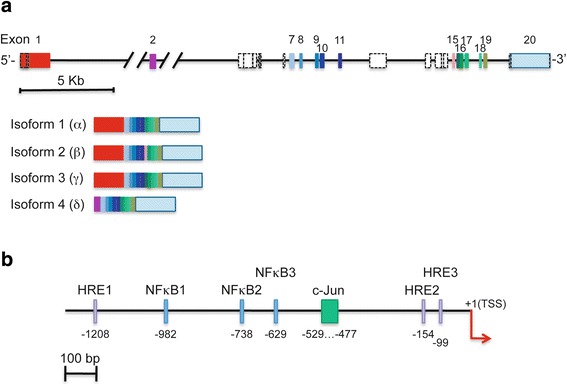
Human paxillin gene map. **a** Exon–intron organization. The gene spans over 55 kb of genomic region in chromosome 12q24 and contains at least 20 exons (shown in *boxes*). Exons contained in isoforms 1–4 are shown in *color*, whereas exons contained in predicted alternative splice variants are shown as *white boxes*. The alternative initiation sites, as well as the alternative 5’ donor sites or 3’ acceptor sites are depicted with *dashed lines*. The assembly of isoforms 1–4 is shown below. The position of the exons, as well as their inclusion in the different isoforms, is shown in Additional file 1. The nucleotide sequence of the human paxillin genomic sequences (Gene ID: 5829) containing exons and introns corresponds to GenBank with accession number NC_000012; REGION: complement (120210439..120265771) GPC_000001304. **b** Promoter region. Ensembl Regulatory Element ENSR00000091396 contains PXN promoter. This region spans 3401 bp region: 1271 bp upstream and 2130 bp downstream the transcription start site (TSS). Only experimentally validated regulatory elements are shown [14–16]

The PXN gene is highly conserved among species: orthologs of human PXN [8] have been identified in 168 organisms [9]. Human PXN gene is located on chromosome 12q24 and contains, at least, 20 exons within its 55 kbp length (Fig. 2a). Four isoforms derived from alternative splicing have been described: Isoform 1(α) represents canonical paxillin [9]. Isoform 2(β) results from the insertion of exon 15, and isoform 3(γ) derives from the use of an alternative 5′-donor site for exon 16. Isoform 4(δ) shares isoform 1 structure, except for a shorter N-terminal domain, derived from the use of an alternative transcription initiation site in exon 2 [10] (Fig. 2). Paxillin isoforms exhibit different expression patterns, α form being the most widely expressed, whereas the expression of the β isoform is more restricted, and that of paxillin γ is related to specific cell differentiation stages [11, 12]. Paxillin δ seems to be exclusively expressed in epithelial cells [10]. Remarkably, at least 26 additional alternative splice variant messenger RNAs (mRNAs) derived from automated computational analysis are predicted in the Genebank (Additional file 1), suggesting that paxillin might have additional isoforms whose functional role remains to be established.

Bioinformatic analysis of PXN promoter region (Ensembl Regulatory Element ENSR00000091396) results in a 3401-bp region, 1271 bp upstream and 2130 bp downstream the transcription start site (TSS), and contains a number of putative binding sites for several transcription factors including AP-1, CREB, STAT3, c-Jun, and c-Myc [13]. The regulatory elements which have been experimentally validated are shown in Fig. 2b [14–16].

MicroRNAs (miRNAs) are small noncoding RNAs that serve as negative regulators of gene expression [17] and have been suggested as key players in the regulation of tumor cell proliferation and invasion [18]. Among these, miR-212, miR-132, miR-145, miR-137, and miR-218 have been shown to target the 3′UTR region of PXN. Furthermore, their levels are inversely correlated with PXN expression in gastric cancer cell lines, colorectal cancer, as well as in non-small cell lung cancer [19–23]. Therefore, the expression of these miRNAs has been proposed as predictive of tumor malignancy and as therapeutic factors.

### Structure of paxillin protein

**Fig. 3:**
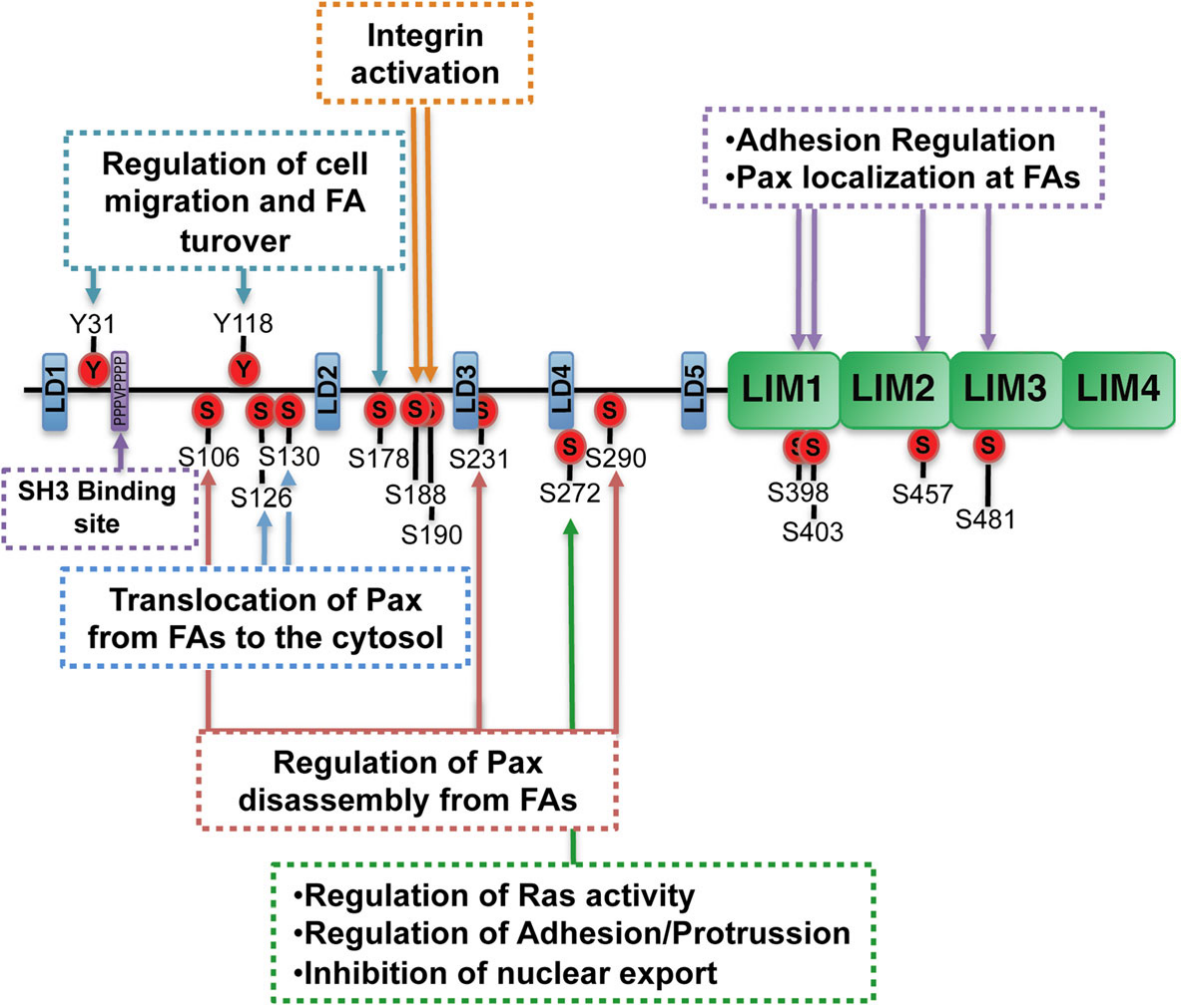
Paxillin structural and functional organization. Human paxillin protein includes 591 amino acids. The N-terminus contains a proline-rich region that anchors SH3-containing proteins and five leucine-rich LD domains (LD1–LD5), which include docking sequences for the recruitment of signaling and structural molecules such as FAK, vinculin, and Crk. The C-terminus contains four cystein-histidine-enriched LIM domains, involved in the anchoring of paxillin to the plasma membrane and its targeting to focal adhesions. Paxillin is phosphorylated in response to cell adhesion and cytokine or growth factor stimulation: Tyrosine phosphorylation generates interaction sites for signaling molecules, which in turn regulate paxillin targeting to FAs. Serine phosphorylation, results in a number of functional modifications involved in the regulation of the adhesion process and cell movement

Paxillin is an adapter protein containing five repetitive leucine-rich LD (Leu-Asp) motifs located at the N-terminus (LD1 to LD5) and four cystein-histidine-enriched LIM domains at the C-terminus. Additionally, it contains a proline-rich sequence that anchors SH3-containing proteins, located within the N-terminus, as well as numerous serine and tyrosine residues throughout the protein, which bind to SH2 domains (Fig. 3).

The LD motifs form amphipathic helices with leucine residues laterally aligned on the helix backbone, thus generating a hydrophobic patch surrounded by negatively charged aspartic and glutamic acid residues [24]. LD domains provide docking sites for FA-associated proteins including tyrosine kinases of the Src family, focal adhesion kinase (FAK), actopaxin, vinculin, talin, integrin-linked kinase (ILK), p21-activated kinase (PAK), as well as the ARF-interacting adapters, G-protein-coupled receptor kinase-interacting proteins (GIT1/GIT2), ADP-ribosylation-interacting protein (ARF), and GTPase-activating proteins (GAPs). FAK interacts with paxillin at an evolutionary preserved LD consensus motif (LDLLXXLL), identified as the binding site for FAK and vinculin [25]. However, in the absence of the four C-terminal LIM domains, the binding of FAK and vinculin to the LD domains is insufficient for directing paxillin to the proper location. In fact, the C-terminal domain of FAK contains a ~150 amino acid “focal adhesion targeting (FAT) region” as well as binding sequences for paxillin and talin. Moreover, although FAK is recruited from the cytoplasm to adhesion sites by paxillin, FAK interaction with N-terminal paxillin domains is not required for paxillin localization at focal adhesions [26]. Particularly, the LD4 domain has been shown to anchor signaling proteins such as GIT1 and paxillin kinase linker p95 in addition to FAK. The proline-rich sequence at the N-terminal also provides a binding site for SH3 domains of the Src family and associates with the tyrosine phosphatase PTP-PEST known to regulate cell spreading and migration [5, 27]. Interestingly, the LD motifs also serve as docking sites for the E6 oncoprotein of bovine papillomavirus type 1, whose binding to paxillin is closely associated with disruption of the actin cytoskeleton and cell transformation by papillomavirus E6 oncoproteins [28]. Noticeably, differences between paxillin isoforms reside at the N-terminal region: Isoforms 2(β) and 3(γ) contain, respectively, 34 and 48 amino acid inserts downstream the LD4 motif [11]. Isoform 4(δ) has a shorter N-terminus than isoform 1(α), lacking the LD1 motif, the proline-rich region, and the key phosphorylation sites Tyr31 and Tyr118 [10].

The C-terminal region of paxillin is common to all isoforms and contains four cystein-histidine-enriched LIM domains, which are involved in protein-protein interactions, and function as an anchor to the plasma membrane. Specifically, the LIM3 domain is responsible for targeting paxillin to FA sites. Of notice, the serine/threonine phosphorylation at LIM2/3 domains cooperatively potentiates the localization of paxillin to FAs (Fig. 3) [26]. Paxillin also associates with the adapter protein 47 (GAG-CRK) [29], a convergence molecule for signals resulting from cell adhesion as well as from the activation of growth factor receptors, which interacts with proteins involved in the regulation of the cytoskeleton and tumor metastasis [27, 30].

## Paxillin phosphorylation

**Fig. 4:**
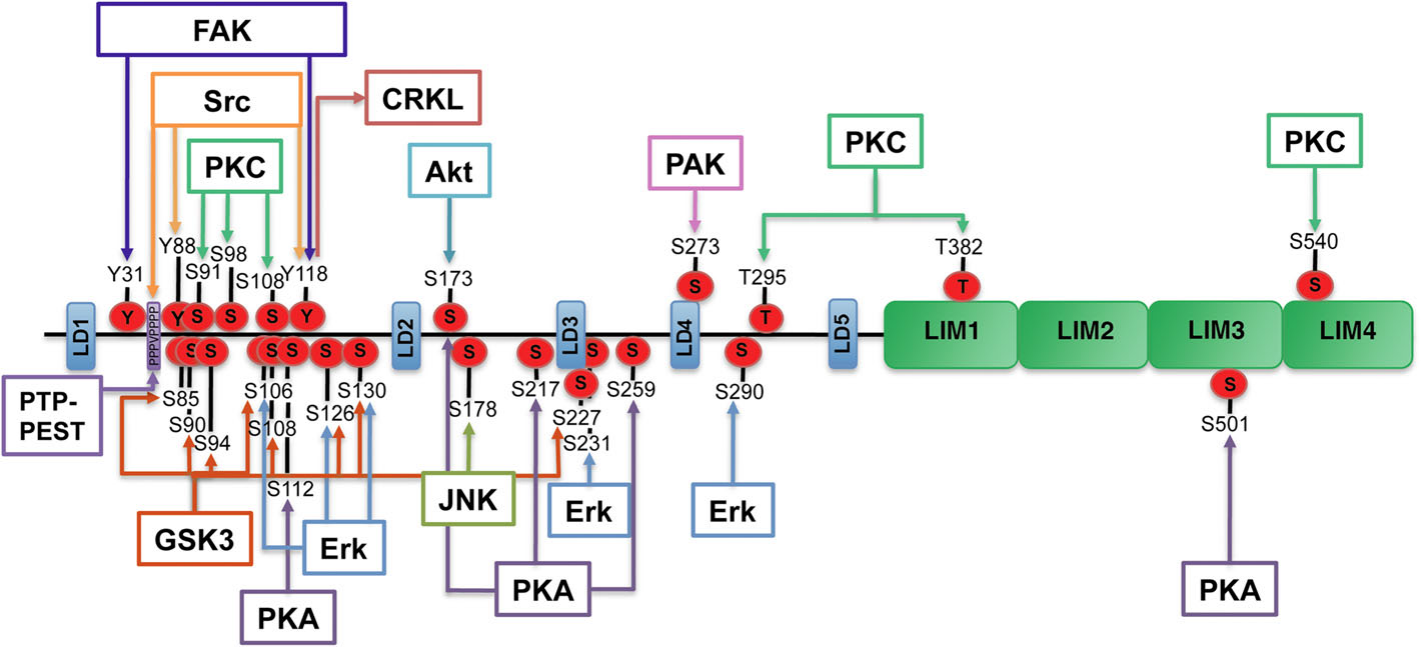
Activation of signaling pathways linked to paxillin phosphorylation. The localization of paxillin and the subsequent recruitment of signaling and adapter proteins are highly dependent on phosphorylation. Tyrosine phosphorylation of paxillin promotes the binding of SH2 domain-containing signaling proteins. In turn, paxillin is also a target for multiple upstream signaling pathways, which upon phosphorylation of specific serine residues modulate the expression, turnover, and intracellular localization of paxillin

The function and localization of paxillin is tightly regulated by phosphorylation [27]. In addition to growth factors and integrin-dependent adhesion to ECM, diverse stimuli have been shown to induce paxillin phosphorylation. Although Tyr31 and Tyr118 are the major phosphorylation sites, paxillin is phosphorylated at multiple Tyr and Ser residues (Figs. 3 and 4) [31].

### Tyrosine phosphorylation

Integrin binding to ECM promotes paxillin phosphorylation and recruitment of proteins involved in the assembly of FAs, including talin, vinculin, tensin, and FAK. Tyr phosphorylation of paxillin provides a scaffold for the recruitment of tyrosine kinases FAK and Src, which are activated as a result of adhesion or growth factor stimulation. The subsequent phosphorylation at Tyr88 and Tyr118 by Src [1, 32], as well as at Tyr118 and Tyr31 at the N-terminus by FAK, allows paxillin interaction with downstream effectors, such as p130cas, and the transduction of external signals into cellular responses mediated by mitogen-activated protein kinases (MAPKs) (Fig. 4). In addition to Src, paxillin Tyr118 binding to CRKL SH-2 domains and thereby to a Rac1 signaling complex activated by DOCK180 has also been described [33]. Although in vitro studies have shown Tyr118 to be the main residue for paxillin phosphorylation by FAK, such phosphorylation is not essential for paxillin localization to focal adhesions. In fact, the binding site for FAK is located at the N-terminal domain, whereas the sequence for localization at focal adhesions resides in the C-terminal region of paxillin. The interaction with FAK however increases the efficiency of paxillin phosphorylation, in addition to directing the phosphorylation of additional tyrosine residues [34]. Since diverse stimuli such as lysophosphatidic acid or platelet-derived growth factor promote Tyr phosphorylation of paxillin and FAK in distinct cell types during spreading, the phosphorylation of paxillin by FAK has been suggested to mediate transduction from cell adhesion to the reorganization of the cytoskeleton required for cell movement [35]. Tyrosine phosphorylation of paxillin has been shown to regulate both the assembly and the turnover of adhesion complexes; however, the significance of this process for adhesion dynamics is still unclear. Whereas phosphorylated paxillin seems to enhance lamellipodial protrusions, non-phosphorylated paxillin has been found essential for fibrillar adhesion formation and fibronectin fibrillogenesis. Recent data show that FAK preferentially interacts with phospho-tyrosine paxillin, which is implicated in high turnover of focal complexes and translocation of FAs [32].

### Serine phosphorylation

The N-terminal domain of paxillin contains a number of serine/threonine phosphorylation sites, which become phosphorylated during cell migration. Recent studies suggest that paxillin turnover, including its degradation by the proteasome, is also regulated by serine phosphorylation during migration. Among the kinases that phosphorylate paxillin at Ser/Thr residues, the phosphorylation of Ser178 by JUN N-terminal kinase (JNK) is required for cell migration [36, 37]. On this line, Ser126 phosphorylation by the sustained activation of Raf/MEK/ERK, and of Ser130 by glycogen synthase kinase 3 (GSK3), has been shown to induce the translocation of paxillin from FAs to the cytosol (Fig. 4) [38]. Furthermore, paxillin phosphorylation at Ser126 and Ser130 has also been found to regulate cytoskeletal rearrangement and cell movement in lipopolysaccharide-stimulated macrophages, through an extracellular regulated kinase (ERK)/GSK3 dual-kinase mechanism, which requires ERK-mediated phosphorylation of Ser130 for the subsequent phosphorylation of Ser126 by GSK3 [33]. Additionally, p21-activated kinase (PAK)-induced phosphorylation of paxillin at Ser273 has been identified as a critical regulator of Rac activation, cell adhesion, and protrusion, through a novel mechanism regulated by FAs [39].

The phosphorylation of serine residues at paxillin LIM domains has been suggested to regulate paxillin localization at FAs, as well as cell adhesion to fibronectin. Integrin binding to ECM promotes the phosphorylation of Ser188 and Ser190, whereas Ser112, 173, 217, and 259 at LD domains and Ser501 at LIM3 are substrates for protein kinase A (PKA). Also, Ser residues 85, 90, 94, 106, 108, 126, and Ser227 at LD3 domain are phosphorylated by GSK3, whereas Akt posphorylates Ser173. The serine residues 398 and 403 at LIM1, 457 at LIM2, and 481 at LIM3 are phosphorylated not only as a consequence of cell adhesion but also by angiotensin II stimulation. Additionally, protein kinase C (PKC) has been found to phosphorylate serine residues 91, 98, 108, 382, and Thr 295 and 540. Moreover, ERK 1/2 which localize to FAs and regulate paxillin disassembly from adhesion complexes, phosphorylate paxillin at Ser106, 231, and 290 [40, 41].

Although the precise involvement of serine phosphorylation in the regulation of adhesion dynamics and migration is not fully understood, the phosphorylation of Ser272 at the LD4 motif seems to regulate the nuclear-cytoplasmic localization of paxillin by blocking the nuclear export of paxillin, thus allowing nuclear accumulation. Recent evidence on this matter shows that nuclear paxillin promotes DNA synthesis and proliferation, and also suppresses H19 gene expression, which is a parental imprinting gene hypothesized to be a tumor supressor [5].

## Integrins and focal adhesions

A number of cell types require integrin-mediated attachment to extracellular matrix (ECM) for proliferation and survival. Integrin binding to ECM results in the Tyr phosphorylation of paxillin and FAK, the formation of FAs, and the assembly of actin fibers required for strenghthening and stabilizing cell interaction with ECM [3, 42]. There are 24 known integrin α subunits and 9 β subunits: the particular combination of two subunits (α, β) generates specific integrin receptors which mediate the binding to the ECM via their extracellular domains, and interact via their cytoplasmic moieties with cytoskeletal adapter proteins such as paxillin [43], and also promote adhesion-mediated signaling through the activation of integrin-associated Tyr kinases, resulting in the phosphorylation of downstream signaling molecules. In this line, recent studies on the hierarchical assembly of FAs have shown that the structure of integrin complexes and the related pathways may vary in different scenarios according to the subunit composition of integrin receptors and the specific signaling pathways activated by distinct combinations [44]. Nevertheless, paxillin recruitment to the leading edge of membrane protrusions is an early event in the formation of focal adhesions [45], although FAK incorporation to these complexes precedes that of paxillin. In fact, the inhibition of FAK/paxillin interaction results in the absence of FAK from focal adhesions, a decrease in the phosphorylation of several adhesion proteins and the consequent alteration of cell migration and invasion (Fig. 3) [3, 46].

## Interaction of paxillin with focal adhesion proteins

The phosphorylation of paxillin at distinct tyrosine and serine residues depends on the cell type and the specific stimulus. Paxillin is a well-known substrate for the FAK/Src complex, which phosphorylates Tyr31 and Tyr118 in dynamic adhesions, thus promoting paxillin disassembly from the adhesion complex. On this line, phosphorylation of Tyr31, Tyr118, Ser188, and Ser190 has been shown to promote migration, suggesting their involvement in adhesion turnover [47]. This assumption is supported by data showing that site-directed mutagenesis of Tyr31/Tyr118 or Ser178 reduces the disassembly of FAs and delays migratory/proliferative phenotype of endothelial cells. In line with these findings, the requirement of FAK and paxillin phosphorylation for insulin growth factor (IGF)-induced cell migration [48] has led to the suggestion that paxillin (together with p38 MAPK activation) could also regulate VEGF-dependent angiogenic response in this cell type [49]. Furthermore, integrin ligand promotion of mouse and human endothelial cell mobility has been shown to involve an ABL1-dependent mechanism, which requires paxillin phosphorylation, the formation of FAs, and the reorganization of the cytoskeleton [50].

### FAK

Paxillin incorporation to FAs is conformationally regulated and is essential for the recruitment of FAK and other scaffolding and signaling molecules involved in FA assembly. The autophosphorylation of FAK at Tyr397 upon integrin activation generates a binding site for the SH2 domain of Src tyrosine kinases, thereby recruiting Src to FAs for the formation of an active FAK/Src kinase complex which phosphorylates distinct focal adhesion proteins including FAK, paxillin and p130cas, leading to the activation of signaling to cell migration [30]. In addition to Src recruitment, FAK has been shown to transmit integrin-stimulated signals to JNK. Recently, paxillin Ser178 has been identified as a major target for JNK, and proven essential for integrin-induced cell movement by means of the JNK–paxillin connection. The activation of JNK by the association of the small GTPase Rac with ERK1/2 and the downstream phosphorylation of paxillin at Ser178 have been proven essential for distinct stages of cell movement and migration [37]. Paxillin phosphorylation also allows binding to the SH2/SH3 domains of CRK, involved in cell mobility through the activation of Rac [29]. On this line, phospho-paxillin binding to the SH2 domain of p120RasGAP results in the activation of p190RhoGAP and the consequent decrease in the activity of RhoA, which could allow cell movement through the disassembly of the actin cytoskeleton [51].

In addition to integrins, a diversity of stimuli including bombesin, lysophosphatidic acid, and PDGF promote paxillin tyrosine phosphorylation by FAK at FAs [35, 42]. Whereas the phosphorylation of paxillin at Tyr118 regulates the affinity of FAK binding to paxillin, the dephosphorylation of phospho-Tyr118 may retard cell migration even when accompanied by increased expression of phospho-FAK Tyr397, which further underlines the importance of paxillin Tyr118 phosphorylation in the full activation of the FAK/paxillin complex and the induction of cell migration.

Of notice, paxillin isoforms exhibit important differences regarding FAK and vinculin binding. Compared to paxillin α, isoform β exhibits reduced in vitro binding to vinculin but equivalent binding to FAK. In contrast, Paxillin γ is defective for FAK binding in vitro but retains vinculin-binding activity. The reason for these differences is not clear, since the insertions do not appear to disrupt the LD4 motif, which binds FAK/vinculin. It is possible that the insertions disrupt interactions with binding partners that stabilize the association of FAK or vinculin with paxillin. Alternatively, these insertions may alter the conformation of the protein masking recognition of both the LD2 and LD4 motifs by FAK or vinculin [11].

### Vinculin

Paxillin interacts with vinculin at FAs through the C-terminal region of vinculin which functions as a binding site for the LD1, LD2, and LD4 domains of paxillin. The high degree of homology of the FAT region of FAK with the C-terminal tail of vinculin suggests that vinculin oligomers could simultaneously bind the LD2 and LD4 motifs, thus interfering with FAK binding to paxillin [52, 53]. Not only vinculin phosphorylation at Tyr1065 regulates its conversion to an open ligand conformation thus allowing binding to talin, F-actin, and other cytoskeletal-related proteins, but it has also been involved in the regulation of apoptosis mediated by the ERK1/2 pathway [54]. Hence, although vinculin and paxillin colocalize at focal adhesions, these two proteins function independently, and vinculin could subserve a broader role in cell cytoskeletal dynamics [55, 56].

### Tubulin

Polarized cell migration is essential for normal organism development and is also a critical component of cancer cell invasion and disease progression. Directional cell motility requires the coordination of dynamic cell–ECM interactions as well as repositioning of the Golgi apparatus, both of which are controlled by the microtubule (MT) cytoskeleton. Dynamic microtubules closely interact with FAs in distinct migrating cells. Examination of microtubule dynamics associated with FAs showed that paxillin acts as an upstream regulator of site-specific microtubule transition from growth to shrinkage (“catastrophe”) at specific, paxillin-rich adhesion domains. Tubulin binds to paxillin LIM2 and LIM3 domains, which requires the integrity of the paxillin zinc finger sequences. Furthermore, replacement of full-length paxillin at adhesion sites by microinjected paxillin LIM2-LIM3 domains suppressed “catastrophe” exclusively at adhesions, suggesting that paxillin regulates microtubule dynamics at adhesion complexes [57].

In addition to its role in FAs, paxillin has been linked to distinct cellular processes in cells lacking these adhesions. In hematopoietic T cells, paxillin is constitutively associated with microtubules and microtubule-organizing centers (MTOC) in a phosphorylation-independent manner. Moreover, paxillin association with the peripheral supramolecular activation center (pSMAC) contributes to the reorganization, docking, and specific location of MTOCs. Along this line, the Src family members Lck (lymphocyte-specific protein tyrosine kinase) and Fyn are key upstream mediators of PI3K and MAPK signaling pathways. As a downstream target of these tyrosine kinases, paxillin has been proposed to contribute to the reorientation and function of the MTOC [24] by recruiting Pyk2 to the MTOC [58]. Paxillin has also been shown to interact with the N- and the C-terminus of γ-tubulin in T lymphocytes, suggesting that paxillin could be responsible for linking microtubule extremes to filamentous actin cortical termini [59].

Recent work has identified a new and conserved role for paxillin in the regulation of the post-translational modification of the MT cytoskeleton, through an inhibitory interaction with the α-tubulin deacetylase HDAC6, mediated by the proline-rich domain of paxillin. Through this interaction, paxillin regulates Golgi organelle integrity as well as polarized cell invasion and migration in normal and transformed cells [60]. Since FAK-mediated activation of RhoA has been shown to stimulate MT acetylation [61], it is plausible that RhoA activation may promote the association of paxillin and HDAC6 and thus result in HDAC6 inhibition and concomitant enhancement of MT acetylation.

## Recruitment of signaling molecules

The protein tyrosine kinase Pyk2 and the tyrosine phosphatase PTP-PEST are known regulators of scaffolding and signaling molecules activated in response to integrin binding. Integrin-induced increase in intracellular calcium promotes Pyk2 autophosphorylation at Tyr402, essential for its catalytic activation and downstream signaling via paxillin, p130cas, Src, Cbl, gelsolin, and PTP-PEST [62]. PTP-PEST is necessary for integrin-mediated adhesion in endothelial cells, normal embryonic development, and mouse embryo viability due to its ability to dephosphorylate cytoskeletal adapter proteins including p130cas, paxillin, and the actin-associated PCH-family-member proline-serine-threonine phosphatase-interacting protein (PSTPIP). Moreover, it associates with the adapters Grb2 and Shc and, through paxillin, with FAK and Pyk2 tyrosine kinases. Suppression of this phosphatase induces an increase in the phosphorylation of its direct substrates p130cas, paxillin, and Pyk2 and causes defects in cell migration and adhesion [63]. Particularly, binding of PTP-PEST to paxillin LIM domain regulates cell spreading, protrusion formation, and migration by dephosphorylating p130cas and decreasing the activity of Rac1 [64]. Direct binding of PTP-PEST to paxillin inhibits the signaling cascades regulated by the LD4 domain [1, 25]. Although paxillin is not a direct target of PTP-PEST, this phosphatase can mediate the dephosphorylation of Tyr residues 31 and 118 of paxillin and the resulting inhibition of Rac, through the dephosphorylation of FAK.

## Recruitment and regulation of monomeric GTPases

The role of paxillin in the promotion of cell migration, fundamental for wound healing, immune response, angiogenesis, and embryogenesis, is exerted through binding to regulators and effecters of the Rho GTPases Rho, Rac, and Cdc42 following integrin–ECM engagement [65].

Within the family of Rho GTPases, Rac and Cdc42 are respectively involved in the formation of lamellipodia and filopodia at the leading edge of migrating cells, whereas RhoA promotes the maturation of adhesions at the leading edge together with their disassembly at the rear of the cell [66, 67]. Paxillin recruitment of the PKL-PIX-PAK complex to FAs may promote the transition from Rho- to Rac-mediated assembly of focal contacts in order to facilitate membrane protrusion and cell migration, suggesting that paxillin may play an important role in regulating signals from Rho GTPases to the actin cytoskeleton [68].

The activity of Rho GTPases is stimulated by guanine nucleotide exchange factors (GEFs) and negatively regulated by GTPase-activating proteins (GAPs) as well as by guanine nucleotide dissociation inhibitors (GDIs) [69], known to play a role in cell migration [70, 71]. Paxillin coordinates the spatiotemporal activation of Cdc42, Rac1, and RhoA GTPases by recruiting GEFs and GAPs along with specific effecter proteins to FAs, thus regulating cell adhesion and spreading during polarized cell migration [66, 67]. Among these regulators, the interaction of ARF-GAP family members with paxillin LD4 domain localizes them to adhesions via Arf-GAP2 [64], which links paxillin to Rho signaling. This interaction is required for the precise coordination of localized Rac1 activity and likely contributes to regulate lamellipodia extension and adhesion turnover by PAK [68]. Paxillin may also regulate Arf GTPase activity through the formation of a complex with the Arf family GEF factor cytohesin-2, mediated by the LIM2 domain of paxillin, which has been shown to regulate migration through Arf6 [72].

## Paxillin function in specific cells and tissues

A number of upstream signals have been shown to activate and regulate paxillin (and FAK) biologic activity, which depends on the cell type, the extracellular stimuli, the intracellular signals activated by stimulating agents, and the metabolic state of the cell (Fig. 4). Among these pathways, PMA-induced ERK activation stimulates phosphorylation of paxillin Ser83 by either ERK or p83, which regulates morphological changes in murine epithelial cells as well as neurite extension in PC12 rat pheochromocytoma cells [37], timocytes and splenocytes [73]. Particularly, the phosphorylation of paxillin at Ser178 carried by JNK has been shown to regulate migration of cells from rat bladder tumors, hamster ovary, and human mammary cancer, as well as the extension of neurites in mouse neuroblastoma cells. In contrast, JNK-mediated paxillin phosphorylation is basally prevented in human epidermal keratinocytes by the blockage of JNK activity by overexpression of a dominant-negative form of JNK1 [74].

### Paxillin in the eye

#### Retina

The cell–cell and cell–extracellular matrix interactions necessary for cell adhesion, migration, proliferation, and differentiation, are essential for the establishment of the stratified cellular organization of the retina during embryonic development [75]. The regulation of this process requires integrin-regulated signaling through paxillin direct binding to the tail regions of α4 integrins and indirect interaction with the tail of β-integrins [76, 77]. On this line, the specific developmental regulation of paxillin Tyr phosphorylation, together with the colocalization of Tyr-phosphorylated paxillin with integrin receptors at adhesion areas in the developing retina suggests the participation of paxillin in the regulation of neuronal migration and neurite extension during retinal development.

Although little is known regarding the possible function of paxillin in the differentiated retina, the radiation-induced activation of paxillin by p38 MAPK in retinal endothelial cells has been shown to trigger cell proliferation and neovascularization which can be prevented through the inhibition of paxillin phosphorylation by the quinic acid derivative KZ-41. This evidence suggests that uncoupling of p38MAPK-mediated signal transduction could be a potential strategy for the design of clinical treatments for radiation retinopathy leading to retinal neovascularization [49].

#### Cornea

The corneal epithelium acts as a protective barrier on the corneal surface and is exposed to physical, chemical, and biological injuries. The successful healing of corneal lesions includes cell migration and proliferation, as well as matrix deposition and tissue remodeling. Among the factors known to promote this process, EGF stimulation of human corneal epithelial cell migration requires the phosphorylation of paxillin by MAPK-JNK at Ser178, which allows the binding of FAK and the subsequent phosphorylation of paxillin at tyrosines 31 and 118 [37]. In fact, ERK1/2 activation in response to a corneal lesion promotes the formation of FAs and the phosphorylation of FAK and paxillin at the border of the wound, which is prevented by MEK1 inhibition. These data clearly involve signaling by ERK/FAK/paxillin in the formation of focal adhesions and the regulation of cell migration associated with corneal wound healing [78]. Recent findings have also revealed that vimentin filaments linked to paxillin-containing FAs at the lamellipodial tips of the cells at the wound edge is essential for wound healing in mock cataract surgery [79].

The phosphorylation of paxillin at Ser178 by c-Jun N-terminal kinase (JNK) has been shown to be vital for epithelial cell adhesion and migration. Studies in human corneal epithelial cells in vivo and in vitro have demonstrated that transglutaminase (TG)-2 positively regulates the phosphorylation of paxillin at Ser178, which does not require stimulation by specific extracellular ligands. Although the signaling mechanism driving Ser178 phosphorylation is poorly explored, paxillin phosphorylation at this residue facilitates the binding of FAK, which in turn promotes Tyr31 and Tyr118 phosphorylation and the subsequent binding of vinculin to paxillin. This evidence suggests that phosphorylation of paxillin Ser178 may be upstream FAK and vinculin, which may be sufficient to interfere with post-translational changes downstream FAK and vinculin [80].

### Immune response and inflammation

The innate immune function of phagocytosis of apoptotic cells, tissue debris, pathogens, and cancer cells is carried out by resident microglia in the central nervous system (CNS). In healthy cells, this process is arrested by the recruitment of immune inhibitory receptor SIRPα to the surface of phagocytes and the resulting activation of signals that inhibit phagocytosis. Recent work showed that the activation of SIRPα prevents paxillin phosphorylation indirectly, through the inactivation of cofilin, induced by paxillin dephosphorylation at Tyr118. These observations support a novel mechanism whereby paxillin and cofilin are targeted to control phagocytosis by phagocytic receptor signaling to the activation of paxillin and cofilin, and signaling by immune inhibitory SIRPα which promotes the inactivation of paxillin and cofilin [81].

Blood leukocytes are involved in essential functions including the development of the immune system and the control of the inflammatory response, which requires migration of leukocytes and lymphocytes from the vasculature, controlled by integrin receptor signaling. Within this process, paxillin binding to α4 integrin cytoplasmic domain induces ligation of integrin α4β1, thus promoting the signaling events. Since paxillin can associate with nonreceptor tyrosine kinases such as FAK and Pyk2, signaling from integrins to these kinases may be implicated in the regulation of leukocyte migration [82, 83].

In inflammatory responses, endothelial cells undergo morphological changes to allow for the passage of neutrophils from the blood vessels to the site of injury or infection. Recent findings showed a selective loss of paxillin and FAK from FAs in proximity to transmigrating neutrophils in HUVEC endothelial cells. As paxillin dynamics are partially regulated by FAK, decreases in FAK protein or disruption of FAK signaling prevents neutrophil transmigration, revealing a novel role for endothelial FA proteins paxillin and FAK in the regulation of inflammation and immune responses [84].

### Epithelial morphogenesis

Paxillin serves as an ERK-regulated scaffold for coordinating FAK and Rac activation, essential for cytoskeleton rearrangement involved in epithelial morphogenesis [40, 85]. The regulation of FA formation/turnover and the interaction of FAs with the actin cytoskeleton are central events for the morphogenic actions of distinct growth factors. Among them, hepatocyte growth factor (HGF) stimulation promotes Src activation and the recruitment of inactive ERK to paxillin, which induces ERK activation. The phosphorylation of paxillin by activated ERK enhances FAK recruitment to nascent adhesions. Ser83 has been identified as the ERK phosphorylation site in murine paxillin, which is essential for HGF-stimulated paxillin–FAK association, FAK phosphorylation, PI3K activation, and the subsequent activation of Rac [47]. This process allows the disassembly of specific adhesions surrounding HGF receptor along morphogenesis, thus promoting lamellipodia formation, cell spreading, and migration [41].

### Embryonic development

Signaling through integrin receptors is importantly involved in developmental processes mainly related to mesodermal-derived structures. Although evidence regarding paxillin role in development is scarce, as a well-known integrin effector, paxillin acts as transducer of fibronectin receptor signals during early development by promoting the recruitment and phosphorylation of FAK, p130cas, and MAPKs [86]. Thus, paxillin and fibronectin seem to regulate some common embryonic developmental events, by controlling FA dynamics, cell migration, and spreading [53, 87]. In support of this assumption, the deletion of the paxillin gene in mouse embryos is lethal. Specifically, phosphorylation of paxillin by PAK (kinase activated by p21) at Ser273 in the chick (Ser272 in human) has been shown to promote the adhesion and migration of non-neural cells [88]. Also, in this line, the activity of dipeptidyl peptidase IV family protease (DPP9) has been shown to be essential for mice neonatal survival. DPP9 gene silencing or specific inhibition was shown to reduce cell adhesion and migration required for early embryonic development by decreasing the phosphorylation of FAK and paxillin [89]. In fact, the death of paxillin null mice embryos after gastrulation derives from defective mesodermal development [77].

Evidence has been provided demonstrating FAK and paxillin involvement in the regulation of cytoskeletal changes fundamental to neuronal development, such as the hormonal modulation of FAK and paxillin in the feminization of the brain. Whereas FAK and paxillin are highly expressed in the hypothalamus of female rats at birth, the male tissue shows low expression, which is significantly increased by the inhibition of aromatase, suggesting that the combined action of androgens and estrogens may be important in paxillin regulation along development [90].

### Paxillin in the nervous system

Integrin-dependent functions, such as adhesion, migration, and proliferation, are critical in the development and the maintenance of the central nervous system [91]. Integrin-activated signaling, including paxillin phosphorylation, is required for the outgrowth of neurites and the formation of the glial scaffold in the brain and also for remodeling and long-term potentiation within synapses. Paxillin involvement in focal adhesion dynamics plays an important role in neurite extension, and is also required for neurite outgrowth, although the molecular mechanism by which paxillin may regulate these processes is poorly understood [92]. Recent studies have shown that neurite outgrowth induced by laminin requires the phosphorylation of FAK and paxillin, mediated by AKT/GSK-3β signaling. This process seems to be essential for brain development, since the knock-out of laminin induces the decrease of FAK and paxillin phosphorylation accompanied by severe anatomic and neuronal morphological defects [93]. In addition to serine phosphorylation, tyrosine phosphorylation of paxillin has also been shown to promote neurite outgrowth induced by NGF and EGF-dependent pathways. Additionally, Nemo-like kinase has been suggested to control the dynamics of the cytoskeleton downstream of NGF signaling, through directly phosphorylating microtubule-associated protein-1B (MAP1B) and paxillin at Ser126 [94]. Furthermore, p38MAPK-mediated phosphorylation of Ser85 on paxillin is also involved in NGF-induced neurite outgrowth in PC-12 cells, since mutation of paxillin Ser 85 significantly inhibits NGF-induced neurite extension. Hence, the phosphorylation of paxillin at Tyr and Ser residues is required for NGF-induced neurite extension, probably through regulating focal adhesion organization [95].

Radial glial cells are known to support neuronal migration along the development of the nervous system. Although little is known regarding the role of paxillin in this process, phosphorylation of paxillin at Ser178 has been shown to play a key role in cell migration in the central and peripheral nervous systems. During development of the peripheral nervous system (PNS), intercellular signaling between neurons and Schwann cells induces the migration of Schwann glial cells along axons, wrapping individual axons to form a myelin sheath. In spite of the considerable clinical importance of myelination, the molecular mechanisms underlying this process in the peripheral nervous system as well as within the central nervous system is largely undefined. Recent data on this issue have shown that JNK binds to the first LD region of paxillin and phosphorylates Ser178 to regulate Schwann cell migration, illustrating that paxillin provides one of the convergent points of intracellular signaling pathways controlling neuronal-mediated Schwann cell migration [96].

Oligodendrocytes (OLs) are the myelinating glial cells of the central nervous system (CNS). Oligodendrocyte precursor cells (OPCs) differentiate into oligodendrocytes (OLs) in order to form the myelin sheath, which wraps axons and allows the rapid propagation of action potentials in the vertebrate nervous system. Studies from Miyamoto et al. [97] have recently shown that cyclin-dependent kinase 5 (Cdk5), a proline-directed serine/threonine kinase enriched in neuronal tissues, directly phosphorylates paxillin at Ser244 following the induction of oligodendrocyte differentiation. Since oligodendrocyte differentiation and myelin sheath formation are prevented by the suppression of Cdk5, paxillin phosphorylation by Cdk5 seems to be a key mechanism in OL differentiation and may ultimately regulate myelination.

## Paxillin in pathological conditions

Numerous pathological conditions may induce the phosphorylation of the focal adhesion partners paxillin and FAK, with the consequent activation of intracellular signals leading to the alteration of cell cytoskeleton, cell division, and cell motility.

### Stress and oxidation

Conditions involving oxidation, mechanical traction, or hypotonic challenge (HTS) have been shown to induce FAK and paxillin phosphorylation and the translocation/activation of RhoA. Athough FAK and paxillin tyrosine phosphorylation is not required for the activation of RhoA/RhoK signaling, the simultaneous activation of RhoA/Rho kinases and FAK/paxillin, driven either by mechanical stress or by receptor activation-induced ATP release, has been found to promote actin cytoskeleton reorganization leading to functional repercussion [98].

Oxidative stress is one of the most common apoptosis-inducing factors in several organs, from the intestinal and cardiovascular systems to neuronal cells, including the sensory organs [99]. In pathological events involving ischemia–reperfusion, reactive oxygen species (ROS), such as superoxide anion (O2·2), hydrogen peroxide (H_2_O_2_), and hydroxyl radical (HO·), are released by the ischemic tissue. ROS, particularly the hydroxyl radical (HO·), are capable of stimulating the tyrosine phosphorylation of paxillin, FAK, and p130cas in human endothelial cells which, in turn, triggers the activation of signaling cascades that regulate cell proliferation and differentiation. Importantly, recent studies show that PAX-FAK-p130cas phosphorylation induced by intracellular hydroxyl radical (HO·) stimulates polymorphonuclear lymphocyte (PMN) adhesion to human endothelial cells and their subsequent migration, suggesting an important role of FAK/PAX activation by ROS in PMN recruitment during inflammation [100].

### Endothelial cell barrier dysfunction

The regulation of paxillin phosphorylation seems to be a target for the control of endothelial cell (EC) barrier function and its dysfunction induced by oxidative stress, as well as lipopolysaccharide- and ventilator-induced lung injury. The release of oxidized phospholipids (OxPLs) into the circulation as a consequence of tissue injury has not only been shown to contribute to pulmonary endothelial cell dysfunction and the alteration of vascular barrier function but can also play a barrier-protective role in human lung ECs through Rac- and Cdc42-dependent promotion of actin reorganization and paxillin accumulation at focal adhesions. Moreover, phosphorylation of paxillin at Ser273 by PAK1 is critically involved in the positive feedback regulation of the Rac-PAK1 pathway, which may contribute to the sustained enhancement of the EC barrier induced by OxPLs [101]. In contrast, paxillin Tyr31 and Tyr118 phosphorylation has been implicated in EC barrier dysfunction causd by OxPL, through the destabilization of VE-cadherin at adherens junctions [1]. Although the opening of endothelial cell junctions causes increased permeability, ECs are capable of enhancing barrier function in the presence of barrier-enhancing factors such as sphingosine-1-phosphate (S1P), hepatocyte growth factor (HGF), and hyperosmolality. This process is driven by HGF- and S1P-dependent modulation of Rac–Rho signaling which, in turn, requires c-Src/FAK-mediated phosphorylation of paxillin at Tyr31 and Tyr118 and also at Tyr181 by c-Abl kinase [102, 103].

### Cancer

Paxillin involvement in cell migration was initially suggested by the high levels of expression of the phosphorylated protein determined in several cancer tissues and metastatic cancer cells, in parallel to increased epithelial-mesenchymal transition [104].

A main characteristic of transformed epithelial cancer cells is the ability to survive and proliferate in the absence of contact with immobilized ECM [105]. Paxillin is known to acquire gain of function mutations that are associated with alterations in the malignant progression of many tumors including breast, lung, prostate, melanoma, and colorectal cancer [106]. Among these, glioblastoma multiforme (GBM), the most common and malignant type of glioma, remains one of the most lethal cancers in the central nervous system (CNS). Recent studies have demonstrated that the overexpression of paxillin at the RNA and protein level is associated with GBM tumor malignancy and hence, predictive of poor survival [107]. These data identify paxillin as a novel prognostic biomarker with potential anti-invasion therapeutic implications in GBM. On this line, a total of 21 unique paxillin mutations were identified in lung cancer tissue specimens and cell lines. A127T, the most common of these mutations, increases tumor growth and invasion in vivo [108]. Importantly, paxillin δ is able to suppress full-length (α)-paxillin signaling as well as interactions with actin-binding and integrin-linked proteins due to the lack of LD1 domain, thus suggesting a potential role in suppressing the migratory phenotype of cancer cells [10].

Recent data show that maximal tyrosine phosphorylation of paxillin by Src and FAK is required for the induction of anchorage-independent signal transduction and proliferation, characteristic of metastatic cells [105, 109]. Along this line, studies by Sero et al. [110] in embryonic fibroblasts (MEF) show that paxillin integrates physical and chemical motility signals by spatially constraining the location of motile processes, thereby regulating directional migration. These findings indicate that complex multimeric and competing interactions on paxillin are required to augment anchorage-independent cell proliferation in cancer development.

A growing number of signaling molecules have been shown to promote or regulate cell migration of cancer cells through the phosphorylation of paxillin at Tyr118 and Ser178 by FAK, which alters the organization of FAs, with the consequent promotion of cell motility [2, 111]. In fact, the increased phosphorylation of these residues is considered as indicative of metastasis.

Multiple receptor-activated signaling pathways have been involved in cell transformation and migration underlying metastasis in breast cancers. Particularly, paxillin has been shown to control the signaling of estrogen (17-β estradiol) to FAK/N-WASP/Arp2/3 complex in breast cancer cells [112]. On this line, the vitamin A derivative retinoic acid (RA), frequently used in cancer therapy, has been shown to induce Src/FAK/PI3K complex signaling to cell attachment, migration, and invasion mediated by the rapid activation of the actin-binding protein moesin. Within this pathway, cell migration of breast cancer cells is prevented by RA through the regulation of the expression of Moesin and the downstream inhibition of Src, FAK, and paxillin activity, providing novel mechanistic clues for the development of new drugs for cancer treatment [113]. Also on this matter, paxillin has been shown to be transcriptionally upregulated and phosphorylated by human epidermal growth factor receptor-2 (HER2) signaling in vitro. Since HER2 overexpression has been involved in metastatic cancer progression, particularly in breast cancer, paxillin has been identified as a marker of aggressive breast cancer and a promoter of neoplastic growth and invasion, supporting its role as a biomarker or therapeutic target [114, 115].

Rho/ROCK signaling plays a crucial role in the regulation of FAs and cell motility [116]. The serine/threonine protein phosphatase inhibitor calyculin A has been shown to promote focal adhesion assembly upon the inhibition of the ROCK downstream target serine/threonine myosin light chain (MLC) phosphatase, and the resulting tyrosine phosphorylation of FAK, paxillin, and p130cas, thus supporting calyculins as potential anticancer agents [117]. The FAK/paxillin pathway regulates small Rho GTPases including RhoA, Rac1, and Cdc42, which are critical determinants of cancer cell migration. Importantly, the specific interaction of Rho GTPases with tumor supressor deleted in liver cancer-1 (DLC1) has been shown to decrease FAK-dependent localization of paxillin at immature focal adhesions, thus controlling the lifetime of nascent focal adhesions [118]. Since this process is FAK-independent in migrated cells, these data indicate that the function of paxillin/FAK/RhoA signaling in the control of cell movement is developmentally regulated. On this matter, the serine/threonine kinase maternal embryonic leucine zipper kinase (MELK) has recently been shown to promote cell migration and invasion via the activation of RhoA and the downstream phosphorylation of FAK and paxillin. Hence, MELK has emerged as a potential cancer biomarker [119]. The human phosphatase and tensin homolog (PTEN) tumor suppressor is mutated at high frequency in a large number of cancers. Recent work in colon cancer cells has shown that PTEN function as a tumor suppressor derives from the downregulation of paxillin expression by the inhibition of PI3K/AKT/NF-kB signaling. This allows the activation of NF-kB and the promotion of paxillin expression, thus stimulating cell invasion, migration, and cancer progression [14]. In agreement with this evidence, proteolysis of paxillin by calpain between the LD1 and LD2 motifs in HeLa cells generates a carboxy-terminal 55 KDa fragment, similar to delta paxillin, which functions as an antagonist of endogenous paxillin and may function to limit cancer cell invasion by preventing the assembly/disassembly of FAs and cell migration [120, 121].

Recent studies in human pancreatic ductal adenocarcinoma cells (PANC-1) have shown that the elevation of cAMP concentration by distinct stimuli results in the inhibition of migration of several PANC cell types in strict correlation with the inhibition of FA turnover, a highly significant loss of paxillin from FAs, and the consequent cessation of ruffling. These data clearly indicate that the inhibitory effect of cAMP on migration, ruffling, FA dynamics, and paxillin localization is mediated by the downstream activation of PKA, while the inhibition of this enzyme potentiates migration [122].

Hepatocyte growth factor (HGF)-induced c-Met signaling plays critical roles in the progression of hepatocellular carcinoma (HCC). On this line, PKCε-mediated c-Met endosomal processing was shown to stimulate c-Met-JNK-induced paxillin (Ser178) phosphorylation, which is required for cell migration, invasion, and intrahepatic metastasis [123].

## Conclusions

Cell migration is a complex process essential for embryonic development, angiogenesis, and wound healing. It involves cell polarization and extension of protrusions and adhesion at the leading edge, coordinated with the disassembly of adhesions at the rear end of the cell. Paxillin has progressively been shown to be crucial for cell movement by recruiting cytoskeletal elements and signaling molecules involved in cell attachment, spreading, and migration. Multiple pathways have been identified which regulate paxillin function through the phosphorylation and dephosphorylation of tyrosine and serine residues. Upon the activation of integrin receptors, FAK phosphorylation of Tyr31 and Tyr118, as well as ERK phosphorylation of serines 106, 231, and 290 have been shown to regulate paxillin disassemby from adhesion complexes and to promote cell movement. On the other hand, binding of PTP-PEST phosphatase was also found to regulate cell spreading, protrusion, and migration. These findings reveal a complex interplay between kinases and phosphatases in the regulation of paxillin function in cell migration. Paxillin has also been shown to coordinate the spatiotemporal activation of Cdc42, Rac1, and RhoA GTPases by recruiting GEFs and GAPs to focal adhesions, thus allowing the activation of a number of cell migration-related signaling pathways. As a major participant in the regulation of cell movement, paxillin plays distinct roles in different cells and tissues, linked to immune response, epithelial morphogenesis, and embryonic development, as well as in pathological conditions such as inflammation, oxidative stress, disruption of endothelial cell barrier, and cancer cell metastasis. The elucidation of paxillin function regulation is crucial for the development of pharmacologic or clinical strategies aimed to controlling cancer development and metastasis.

## Additional file

Additional file 1: Alternative splice variants of human paxillin. The human paxillin gene (Gene ID: 5829) is 55333 bp. “Homo sapiens chromosome 12, GRCh38.p7 Primary Assembly,” NCBI Reference Sequence: NC_000012.12 was used in the elaboration of this table. Alternative splice variants are organized in columns. The putative exons are boxed and shown in the first column. The position of 5’-donor sites, as well as 3’-acceptor sites, is shown in the second column. (XLSX 54 kb)

## Abbreviations

3’-UTR: 3’-Untranslated region
ABL1: ABL proto-oncogene 1 non-receptor tyrosine kinase
ADP: Adenosine diphosphate
AKT: Serine/threonine kinase
ARF: ADP-ribosylation factor
Arf2: Arf family GTPase
Asp: Asparagine
cAMP: Cyclic AMP
Cas: Cas family scaffolding protein
Cdc42: Cell division cycle 42
cDNA: Complementary DNA
CNS: Central nervous system
Crk: Crk adapter protein
Csk: C Src tyrosine kinase
DLC1: Tumor supressor deleted in liver cancer, 1
DOCK 180: Dedicator of cytokinesis
DPP9: Dipeptidyl peptidase IV family protease
EC: Endothelial cell barrier
ECM: Extracellular matrix
EGF: Epidermal growth factor
EPAC: Induced exchange protein activated by cAMP
ERK: Extracellular regulated kinase
FA: Focal adhesion
FAK: Focal adhesion kinase
FAT: Focal adhesion targeting region
Fyn: Src family tyrosine kinase
GAPs: GTPase-activating protein
GDIs: Guanine dissociation inhibitors
GEFs: Guanine nucleotide exchange factors
GIT1/2: G-protein-coupled receptor
Grb2: Growth factor receptor-bound 2
GSK: Glycogen synthase kinase
H_2_O_2_: Hydrogen peroxide
HCC: Hepatocellular carcinoma
HDAC6: Alpha tubulin deacetylase
HER2: Human epidermal growth factor receptor 2
HGF: Hepatocyte growth factor
Hic5: Transforming growth factor beta-1-induced transcript
HIF-1α: Hypoxia-inducible factor 1-alpha
HO·: Hydroxyl radical
HRE: Hypoxia response element
HTS: Hypotonic challenge
HUVEC: Human umbilical vein endothelial cells
IGF: Insulin growth factor
ILK: Integrin-linked kinase
JNK: JUN N-terminal kinase
Lck: Lymphocyte-specific protein tyrosine kinase
Leu: Leucine
LPA: Lisophosphatidic acid
LPS: Lipopolysaccharide
MAPKs: Mitogen-activated protein kinases
MEK: Mitogen-activated protein kinase
MELK: Maternal embryonic leucine zipper kinase
miRNAs: MicroRNAs
MLC: Serine/threonine phosphatase
MT: Microtubule
MTOC: Microtubule-organizing center
NF-κB: Nuclear factor kappa B
O2·2: Superoxide anion
OxPLs: Oxidized phospholipids
P120RasGAP: Ras p21 protein activator
P130Cas: Cas family scaffolding protein
p38: Mitogen-activated protein kinase 14
PAK: p21-activated kinase
PANC1: Human pancreatic ductal adenocarcinoma cells
PDGF: Platelet-derived growth factor
PI3K: Phosphatidylinositol 3 kinase
PIX: Rho guanine nucleotide exchange factor
PKA: Protein kinase A
PKC: Protein kinase C
PKL: Pyruvate kinase liver and RBC
pSMAC: Supramolecular activation center
PSTPIP: Proline-serine-threonine phosphatase-interacting protein
PTEN: Human phosphatase and tensin homolog
PTP-PEST: Protein tyrosine phosphatase-PEST
PXN: Paxillin gene
Pyk2: Protein tyrosine kinase 2
RA: Retinoic acid
Rac1: Ras-related C3 botulinum toxin substrate 1
Raf: Raf kinase
RhoA: Ras homolog family member A
ROCK: Rho kinase
ROS: Reactive oxygen species
RPE: Retinal pigmented epithelium
S, Ser: Serine
S1P: Sphingosine-1-phosphate
Shc: Shc adapter protein
SIRPalfa: Signal regulatory protein alpha
Src: SRC proto-oncogene non-receptor tyrosine kinase
Thr: Threonine
TSS: Transcription start site
Tyr: Tyrosine
VEGF: Vascular endothelial growth factor
WASP: CG 1520 gene product from transcript CG 1520-RA
Y-Tyr: Tyrosine
